# Neutrophil-derived ROS as a rapid functional biomarker: diagnostic and prognostic performance of the Leukocyte ImmunoTest in infection and sepsis

**DOI:** 10.3389/fimmu.2026.1829226

**Published:** 2026-06-29

**Authors:** Nazlıhan Boyacı Dündar, David Sarphie, Kenan Yüce, Gülbin Aygencel, Melda Türkoğlu, Rubina Mian, Paul Moss, Mustafa Necmi İlhan, Gülendam Bozdayı

**Affiliations:** 1 Department of Internal Medicine, Division of Intensive Care Medicine, Gazi University Faculty of Medicine, Ankara, Türkiye; 2 Seroxo Limited, Oxford, United Kingdom; 3 Department of Clinical Microbiology, Division of Virology, Gazi University Faculty of Medicine, Ankara, Türkiye; 4 Institute of Immunology and Immuno-therapy, University of Birmingham, Birmingham, United Kingdom; 5 University Hospitals Birmingham NHS Foundation Trust, Birmingham, United Kingdom; 6 Department of Public Health, Faculty of Medicine, Gazi University, Ankara, Türkiye

**Keywords:** bedside diagnostics, biomarkers, critical illness, infection, Leukocyte ImmunoTest, neutrophil-derived reactive oxygen species (ROS), sepsis

## Abstract

**Background:**

Early diagnosis of sepsis remains a major clinical challenge due to the dynamic interplay between infection and host immune response. Conventional biomarkers often fail to capture the dynamic nature of immune activation. Neutrophil-derived reactive oxygen species (ROS), central to antimicrobial defense and tissue injury, may offer early insight into immune dysregulation. The Leukocyte ImmunoTest (LIT) is a rapid, bedside assay that quantifies neutrophil ROS production within minutes, providing a functional snapshot of innate immunity.

**Methods:**

This prospective observational study was conducted in intensive care and internal medicine wards of a university hospital. Participants were categorized *post hoc* into three groups: inpatient controls (n=29), infection (n=47), and sepsis (n=106). LIT was performed on whole blood samples, expressed as relative light units (RLU), and compared with C-reactive protein (CRP), procalcitonin (PCT), white blood cell count (WBC), and neutrophil count (PMNL). Diagnostic performance was evaluated using receiver operating characteristic (ROC) analysis, and longitudinal LIT trends were assessed in relation to survival.

**Results:**

Median LIT values increased across diagnostic groups: 470 RLU in controls, 882 in infection, and 2466 in sepsis (adjusted p < 0.05). LIT demonstrated good diagnostic performance in identifying infection (AUC: 0.94, 95% CI: 0.911–0.968) and sepsis (AUC: 0.86, 95% CI: 0.795–0.915), with performance comparable to CRP and PCT, respectively. In a joint model, higher LIT values were independently associated with increased mortality (HR:1.6, 95% CI:1.2–2.2; p=0.005).

**Conclusion:**

LIT is a rapid bedside immune assay capturing the dynamic nature of neutrophil activation, demonstrating diagnostic and prognostic performance comparable to established biomarkers such as CRP and PCT in infection and sepsis. These findings suggest that LIT may have potential as a complementary biomarker in sepsis management. Multicenter studies are needed to confirm its integration into sepsis protocols and to further clarify its role in early recognition, risk stratification, and individualized care.

## 1 Introduction

Sepsis is a life-threatening syndrome characterized by severe organ dysfunction due to a dysregulated host response to infection, posing an immense global health burden with an estimated 48.9 million cases and 11 million deaths annually (1, 2). Despite advances in critical care, early diagnosis remains a major clinical challenge due to the heterogeneity of the patient population and the dynamic nature of the immune response (3, 4). Diagnosis relies on clinical scoring systems such as the Sepsis-related Organ Failure Assessment (SOFA), which reflects downstream organ dysfunction rather than upstream immune dysregulation (1, 3). Given the time-sensitive nature of therapeutic interventions, there is a critical need for biomarkers that can accurately reflect the dynamic nature of immune activity during the early stages of the syndrome and facilitate the prompt recognition of sepsis (5).

In sepsis pathophysiology, the host immune system initially mounts a robust inflammatory response through the activation of innate immunity, followed by compensatory anti-inflammatory and immunosuppressive phases (6, 7). Neutrophils, the most abundant circulating leukocytes, serve as the first line of defense against invading pathogens (3, 7). They eliminate pathogens via phagocytosis, degranulation, and the production of reactive oxygen species (ROS), which are generated primarily through nicotinamide adenine dinucleotide phosphate (NADPH) oxidase-mediated respiratory bursts (8). While ROS is essential for pathogen killing, excessive or dysregulated ROS production contributes to oxidative tissue injury and organ dysfunction (7, 9). ROS generation can activate granular proteases and induce the formation of neutrophil extracellular traps (NETs) (7, 8). Both experimental and clinical studies consistently implicate neutrophil-derived ROS in redox-mediated cellular damage during sepsis. Beyond their cytotoxic effects, ROS also amplify inflammation by altering redox balance and modulating key signaling pathways, underscoring their role as critical mediators of sepsis progression (10, 11).

In recent years, various biomarkers have been explored for early sepsis detection, each reflecting distinct aspects of the host-pathogen interaction, such as immune activation and systemic inflammation (12–16). However, their clinical applicability remains limited due to issues of specificity, often tied to a specific pathophysiologic process, as well as delayed kinetics, cost, and accessibility (17). Among emerging host response biomarkers, pancreatic stone protein (PSP) has gained attention as a potential point-of-care marker reflecting early systemic inflammatory activation. Recent studies suggest that PSP may offer favorable discriminative performance for sepsis, although the current evidence base remains relatively limited and further validation across diverse clinical settings is required (18, 19). Additionally, while “omics” technologies show promise in identifying molecular signatures and patient endotypes, their integration into routine practice is constrained by high costs and complex infrastructure requirements (20, 21).

In this context, the Leukocyte ImmunoTest (LIT; Seroxo Ltd., Oxford, UK) offers a novel functional approach by quantifying neutrophil-derived ROS production at the bedside within 10 minutes from a small blood sample (22). Unlike conventional biomarkers that reflect systemic inflammation and provide static concentration data, LIT reflects the dynamic functional capacity of immune cells, offering a potentially more direct link to disease pathophysiology. Our previous study demonstrated the prognostic value of LIT in patients with severe COVID-19, while others have reported higher ROS production in non-COVID sepsis, supporting the test’s broader relevance in critical illness (23, 24). Notably, LIT is not only a functional immune assay but also a rapid and accessible bedside tool that captures the functional status of immune cells, setting it apart from most currently used diagnostic modalities.

Building upon these observations, we conducted a prospective observational study to examine the diagnostic relevance of leukocyte-derived ROS production across a spectrum of patients with and without infection. Specifically, we aimed to determine whether LIT values differ significantly between infected and non-infected patients, and whether ROS production is associated with clinical outcomes, such as survival, within the infection cohort. This is the first study to systematically evaluate the diagnostic performance of LIT in distinguishing infection and sepsis from non-infectious conditions within a prospective cohort, while also proposing preliminary threshold values for potential bedside application.

## 2 Methodology

### 2.1 Ethical approval and patient selection

This prospective observational study was conducted with the approval of the Clinical Research Ethics Committee of Gazi University (Approval Number: 531, Date: 27.06.2022) and was registered in ClinicalTrials.gov (NCT05968287). The study was performed in the 16-bed medical intensive care unit (ICU) and the 7-bed internal medicine ward of Gazi University Hospital, a tertiary academic center in Ankara, Turkey. Written informed consent was obtained from all participants or, in cases of impaired consciousness or clinical instability, from their legal guardians, by institutional ethics protocols and the Declaration of Helsinki. This approach was pre-approved by the ethics committee and ensured the inclusion of a representative sample of critically ill patients, thereby preventing the exclusion of the sickest individuals.

The study population consisted of patients admitted to the medical ICU or inpatient ward due to various infectious diseases, with or without sepsis. The control group consisted of voluntary participants, including outpatients and inpatients, admitted for non-infectious conditions. Outpatients were recruited through invitation during routine clinic visits and enrolled after informed consent. Not all eligible individuals consented to participate, reflecting the voluntary nature of outpatient recruitment in routine clinical practice.

In the control group, LIT and complete blood count (CBC) measurements were performed once on the day of enrollment. Any concurrent infection in control patients was ruled out based on medical history, physical examination, white blood cell count (WBC), and C-reactive protein (CRP) levels obtained on the same day. Procalcitonin (PCT) was not routinely measured in outpatient settings at our institution for the exclusion of infection; it was obtained only at the discretion of the treating physician, and such measurements were recorded when available.

Patient enrollment and follow-up were completed in accordance with the ClinicalTrials.gov protocol, with all participants monitored until death or hospital discharge for inpatients. Infection was diagnosed by the attending physicians based on compatible clinical signs and symptoms, radiological evidence, and/or microbiological confirmation, by institutional protocols aligned with institutional protocols aligned with international guidelines (e.g., recommendations from the Infectious Diseases Society of America and the European Society of Clinical Microbiology and Infectious Diseases). Sepsis and septic shock were defined according to the Third International Consensus Definitions (Sepsis-3), requiring a suspected or confirmed infection together with an acute increase in SOFA score of ≥2 points (25). In cases where the diagnosis of infection or sepsis was not definitive at the time of enrollment, a *post hoc* adjudication was performed at 72 hours by two independent intensive care physicians, who were blinded to LIT values, based on the patient’s clinical evolution and available diagnostic findings. Final classification was determined by consensus.

Patients were excluded if they (i) died within the first 24 hours of hospitalization, as LIT measurements and key diagnostic tests could not be reliably performed within this time frame, and a definitive diagnosis of infection and/or sepsis could not be established due to incomplete clinical and laboratory evaluation in rapidly deteriorating patients; (ii) had an absolute polymorphonuclear leukocyte (PMNL) count <500/mm³ at admission, (iii) experienced a rapid decline in PMNL count to <2000/mm³ within the first 48 hours, given the potential impact of severe neutropenia on LIT measurements or (iv) declined to participate. Additionally, patients with severe systemic inflammatory response syndrome (SIRS) due to non-infectious etiologies, including inflammatory bowel disease, active inflammatory arthritis, and newly diagnosed metastatic cancer, were excluded. SIRS was defined according to the original consensus criteria, requiring the presence of at least two of the following: body temperature >38 °C or <36 °C, heart rate >90 beats/min, respiratory rate >20 breaths/min or PaCO_2_ <32 mmHg, and WBC>12,000/mm³, <4,000/mm³, or >10% immature (band) forms (26). In repeated hospitalizations, only data from the first admission were considered. Outpatients with stable chronic infectious diseases, such as viral hepatitis or tuberculosis, were also excluded from the study.

### 2.2 Patient grouping

Initially, patients were stratified into five groups, based on admission setting and infection status, as outpatient controls; inpatient controls; infection without sepsis; sepsis; and septic shock. This initial five-group classification was used for exploratory purposes to better characterize the heterogeneity of the study population. However, preliminary analyses revealed that outpatient controls exhibited distinct inflammatory profiles compared to inpatient controls without infection. In addition, since septic shock is considered a circulatory failure subtype of sepsis according to Sepsis-3 criteria, and because patients were not always referred to the ICU in the early hours of sepsis, we combined sepsis and septic shock into a single clinically meaningful category. Furthermore, outpatient controls contributed only a single LIT measurement and were therefore not included in longitudinal analyses evaluating LIT trajectories. Therefore, to align the grouping with clinically meaningful patient categorization used in real-world hospital practice, we restructured the primary analysis into inpatient controls, infection without sepsis, and a combined sepsis group (sepsis or septic shock). This grouping approach was consistent with the predefined study design, which specified two main cohorts (infection vs. control) with additional stratification according to sepsis status.

The original five-group analysis was retained as a predefined subgroup analysis and is provided in the **Supplementary Material**.

### 2.3 Measurements of ROS production by luminometer

ROS production was measured in freshly obtained whole blood samples and analyzed within 30 minutes of collection. Briefly, 10 µL of blood, obtained via peripheral venepuncture or from existing intravascular lines using heparinized (green-top) tubes, was added to commercially prepared freeze-dried LIT reagents reconstituted in phosphate-buffered saline (PBS). The reagents consisted of a 10^-5^ M phorbol 12-myristate 13-acetate (PMA; P1585, Sigma) solution and a 1.12 mM luminol (A4685, Sigma) solution in PBS, which were combined and freeze-dried overnight in individual aliquots.

Following reconstitution with 100 µL PBS (0.1×) and addition of the blood sample, the reaction mixture was incubated at 37.5 °C for 10 minutes. Chemiluminescence was then measured using a handheld luminometer (Clean-Trace NG3, 3M) and expressed in relative light units (RLU). Previous assessments of unstimulated luminol oxidation (in the absence of PMA) in healthy volunteers have consistently demonstrated negligible signals (<5 RLU) (27). Accordingly, reported LIT values represent the net chemiluminescent response attributable to PMA-stimulated oxidative activity. Single LIT measurements were made either daily or every other day (as per the study protocol). All measurements were performed under standardized conditions by trained personnel, following the manufacturer’s protocol. Quality control procedures included pre-study instrument calibration and verification using control samples with predefined acceptance criteria as well as reagent quality assurance validation.

The LIT assay provides a quantitative measure of neutrophil-derived ROS production and was used to evaluate whether elevated ROS generation is associated with the presence and severity of infection. LIT values typically range from 1 to 15,000 RLU, with higher values indicating increased ROS production.

### 2.4 Data collection

For the control group, the LIT and CBC were each performed once on the day of the outpatient visit. Any concurrent infection in control patients was ruled out based on medical history, physical examination, WBC, and CRP levels obtained on the same day.

Basic demographic information, including age, sex, reason for hospital admission, and comorbidities, was recorded for all participants. In evaluating the association between LIT values and infection, sepsis, or septic shock, PCT and CRP levels measured on the corresponding study day (if available) for inpatients, were also analyzed for clinical relevance.

For critically ill patients, the Acute Physiology and Chronic Health Evaluation II (APACHE II) and SOFA scores were documented at the time of ICU admission. The primary care team performed routine laboratory investigations to assess the patient’s underlying conditions. Additionally, data regarding the need for organ support, including supplemental oxygen therapy, mechanical ventilation, vasopressor therapy, and renal replacement therapy (RRT), as well as the presence of acute kidney injury (AKI) during the ICU stay, were collected.

### 2.5 Study outcomes

The primary outcomes of the study were defined as follows: (1) to evaluate differences in ROS production, as measured by LIT and expressed in RLU, between patients with infectious conditions (i.e., infection and sepsis) and those without evidence of infection (inpatient control). In addition, the original five-group analysis was retained as a predefined subgroup analysis to explore whether baseline LIT values differ between hospitalized inpatients without infectious diseases and voluntary outpatient controls. This additional comparison aimed to assess potential physiological variability in ROS production unrelated to infection and further validate the diagnostic value of LIT in distinguishing infectious from non-infectious states.

The secondary outcomes were defined as to investigate the association between ROS production levels and clinical outcomes in patients with infection, specifically by comparing LIT values between survivors and non-survivors.

### 2.6 Statistical analysis

Continuous variables are presented as means ± standard deviation (SD), medians with interquartile ranges (Q1–Q3), and categorical variables as frequencies and percentages. Group comparisons were performed using the Kruskal–Wallis test for non-parametric data, with *post hoc* pairwise analyses conducted using Dunn’s test and Benjamini–Hochberg correction for multiple testing. Categorical variables were compared using the Chi-squared or Fisher’s exact test, as appropriate. Receiver operating characteristic (ROC) curve analysis with area under the curve (AUC) estimation and DeLong’s test were used to assess and compare the diagnostic performance of LIT and other inflammatory markers. Spearman’s correlation analysis was applied to evaluate relationships between continuous variables.

Survival analysis was performed using a joint model that combined a Cox proportional hazards model with a linear mixed-effects model, where log-transformed LIT values were included as time-dependent covariates. In exploratory analyses, clinically relevant covariates including age, sex, and comorbidities were evaluated but were not retained in the final model, as they did not significantly improve model fit.

A two-sided p-value <0.05 was considered statistically significant. All analyses were conducted using R (version 4.4.1) and IBM SPSS Statistics (version 22.0). The statistical workflow was developed with a biostatistician team to ensure adherence to contemporary standards of analytical rigor, transparency, and reproducibility in biomedical research. Additional details of statistical methods are available in the **Supplementary Methods** section.

## 3 Results

### 3.1 Baseline clinical characteristics and distribution of inflammatory markers across study groups

**Figure 1:**
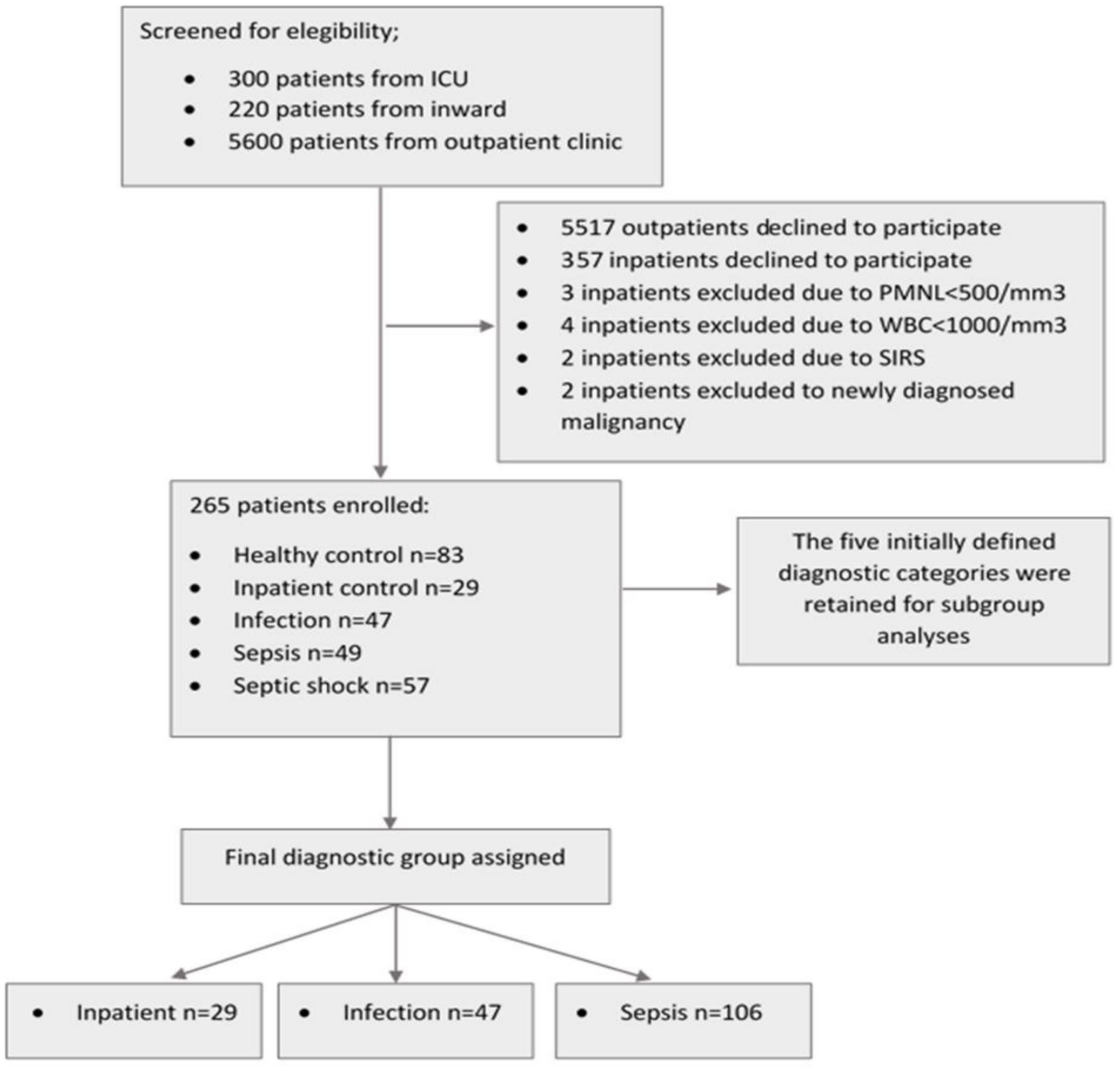
Flow diagram of patient selection and group allocation.

A total of 6,120 patients were screened for eligibility, including 300 critically ill patients, 220 inpatients from internal medicine wards, and 5,600 individuals from outpatient clinics. Among these, only 83 outpatients and 193 inpatients voluntarily agreed to participate in the study. Of those who consented, 3 were excluded due to neutrophil counts <500/mm³, 4 due to white blood cell counts <1,000/mm³, 2 for meeting SIRS criteria without confirmed infection, and 2 due to newly diagnosed malignancies. In total, 265 patients were included in the study and classified into three primary diagnostic groups based on their clinical status and infection severity: inpatient controls without infection (n=29), infection without sepsis (n=47), and a combined sepsis group including both sepsis and septic shock (n=106). The patient selection and grouping process is summarized in **Figure 1**. Baseline demographic characteristics, comorbidities, laboratory findings at admission, ICU course–related features and scores at admission, and the need for organ support are summarized in **Table 1**. The three groups were generally comparable in terms of age and most comorbid conditions, with no statistically significant differences observed. Malignancy was more frequent in the sepsis group compared to the other groups (p < 0.001). Pneumonia was more frequently identified as the source of infection in the sepsis group compared to the infection group (p < 0.001). Laboratory findings demonstrated a progressive increase in inflammatory markers, including WBC and PMNL counts, from controls to infection and sepsis. Lymphocyte count showed a corresponding decrease (all p < 0.001). Parameters associated with organ dysfunction, including BUN, creatinine, bilirubin levels, and severity scores (APACHE II and SOFA), were significantly higher in the sepsis group (all p < 0.05). In addition, the need for invasive mechanical ventilation (IMV) and the incidence of AKI were more frequent in patients with sepsis, consistent with greater disease severity.

**Table 1:** Clinical characteristics and baseline laboratory findings of the study participants.

Variables	Inpatient control (n=29)	Infection (n=47)	Sepsis (n=106)	p-value
**Age, mean (± SD)**	64 ± 19	62 ± 22	71 ± 13	0.120
**Gender, n(%)**				0.075
Female	18 (62.1)	22 (46.8)	41 (38.7)	
Male	11 (37.9)	25 (53.2)	65 (61.3)	
Comorbidities, n (%)				
Hypertension	17 (58.6)	20 (42.6)	53 (50.0)	0.390
Malignancy	1 (3.4)^b^	8 (17.0)^c^	44 (41.5)^b,c^	**<0.001**
Cardiovascular disease	16 (55.2)	16 (34.0)	43 (40.6)	0.187
Respiratory disease	8 (27.6)	13 (31.7)	33 (31.1)	0.923
Diabetes mellitus	13 (44.8)	17 (36.2)	31 (29.2)	0.262
Neurological disease	4 (13.8)	10 (21.3)	26 (24.5)	0.462
Renal disease	5 (17.2)	5 (10.6)	17 (16.0)	0.635
Other endocrinological disease	7 (24.1)	7 (14.9)	14 (13.2)	0.350
GIS diseases	3 (10.3)	2 (4.3)	11 (10.4)	0.444
Rheumatological disease	2 (6.9)	2 (4.3)	11 (10.4)	0.428
Reason for admission, n (%)				
Infection	–	47 (100)	–	NA
Sepsis	–	–	106 (100)	NA
Pulmonary	8 (27.6)	13 (31.7)	33 (31.1)	0.923
Renal	9 (31)^b^	6 (14.6)	10 (9.4)^b^	**0.013**
Neurological	2 (6.9)	3 (7.3)	7 (6.7)	0.988
Cardiac	1 (3.4)	3 (7.3)	7 (6.7)	0 780
GIS disorders	10 (34.5)^b^	6 (14.6)^c^	5 (4.7)^b,c^	**<0.001**
Oncological	4 (13.8)	–	5 (4.8)	**0.035**
Surgical	–	–	4 (3.8)	NA
Metabolic	1 (3.4)	4 (9.8)	3 (2.8)	0.186
Hematological	2 (6.9)	–	2 (1.9)	0.350
Intoxication	–	–	1 (1.0)	NA
Source of infection, n (%)				
Pneumonia	–	9 (19.1)	60 (56.6)	**<0.001**
Urinary infection	–	14 (29.8)	32 (30.2)	0.960
Blood-stream infections	–	6 (12.8)	23 (21.7)	0.193
Intraabdominal infections	–	5 (10.6)	9 (8.5)	0.763
Upper respiratory tract infection	–	10 (21.3)	6 (5.7)	**0.008**
Acute gastroenteritis	–	4 (8.5)	1 (0.9)	**0.031**
Others	–	6 (12.8)	9 (8.5)	0.395
Identified pathogens, n (%)				
Bacterial	-	21 (44.7)	67 (63.2)	**0.032**
Gram (+)	-	6 (12.8)	28 (26.4)	0.061
Gram (-)	-	15 (31.9)	49 (46.2)	0.098
Viral	–	14 (29.8)	22 (20.8)	0.224
Fungal	–	1 (2.1)	11 (10.4)	0.106
Baseline laboratory findings on admission, median [Q1-Q3]				
Hgb (gr/dL)	11.4 [9.2-12.1]	11.7 [9.7-12.7]^c^	10.2 [8.8-12.8]^c^	**0.044**
WBC (/µL)	8400 [6500-10800]^b^	9900 [6800-11400]^c^	13300 [8800-18300]^b,c^	**<0.001**
PMNL (/µL)	5700 [4000-8600]^b^	7400 [4600-9400]^c^	11000 [6700-16100]^b,c^	**<0.001**
Lymphocyte (/µL)	1500 [900-2300]^a,b^	1000 [500-1600]^a^	700 [400-1200]^b^	**<0.001**
PLT (x10.e^3^/µL)	210 [161-319]	227 [173-311]^c^	177 [110-284]^c^	**0.030**
ALT (U/L)	24 [10-72]	21 [13-35]	25 [15-58]	0.308
AST (U/L)	25 [16-62]	23 [15-55]	38 [21-67]	**0.041**
LDH (U/L)	200 [190-470]	295 [191-375]	341 [205-531]	0.056
BUN (mg/dL)	24 [14-45]^b^	29 [15-41]^c^	38 [28-59]^b,c^	**<0.001**
Creatinine (mg/dL)	1.0 [0.64-1.83]^b^	0.99 [0.76-1.72]^c^	1.71 [0.9-2.55]^b,c^	**0.022**
T.Bil (mg/dL)	0.96 [0.55-1.09]	0.66 [0.46-1.0]^c^	1.0 [0.54-1.7]^c^	**0.013**
D. Bil (mg/dL)	0.19 [0.1-0.8]	0.16 [0.1-0.26]^c^	0.3 [0.16-0.8]^c^	**<0.001**
Respiratory support, n (%)				
Nasal oxygen	5 (17.2)	15 (31.9)	42 (39.2)	0.083
HFNO	1 (3.4)	2 (4.2)	10 (9.4)	0.458
Requirement of NIMV	2 (6.9)	3 (6.3)	10 (9.4)	0.646
Requirement of IMV	4 (13.8)	2 (4.3)^c^	28 (26.4)^c^	**0.011**
**Requirement of vasopressors, n (%)**	–	–	57 (53.8)	NA
**AKI, n (%)**	9 (31.0)^b^	10 (21.3)^c^	60 (56.6)^b,c^	**<0.001**
**APACHE-II score, median [Q1-Q3]**	15 [14-24]^b^	14 [12-20]^c^	23 [19-29]^b,c^	**<0.001**
**SOFA score, median [Q1-Q3]**	4 [2-7]^b^	4 [2-5]^c^	7 [4-10]^b,c^	**<0.001**
**LOS, median [Q1-Q3]**	7 [5-20]^b^	10 [7-17]^c^	17 [10-31]^b,c^	**0.003**

p-values refer to the Chi-squared test, Fisher’s exact test or Kruskal-Wallis test across all three groups. Bold values indicate statistical significance (p < 0.05).

Superscript letters indicate significant differences in pairwise comparisons after Benjamini–Hochberg correction: groups with different superscript letters differ significantly (adjusted p < 0.05).

**^a^** inpatient control vs infection.

**^b^** inpatient control vs sepsis.

**^c^** infection vs sepsis.

AKI, Acute kidney injury according to AKIN classification; ALT, Alanine aminotransferase; APACHE, Acute physiology and chronic health evaluation, AST, Aspartate aminotransferase; D.Bil, Direct bilirubin; BUN, Blood urea nitrogen; CRP, C-reactive protein; GIS, Gastrointetsinal; Hgb, Hemogloblin; HFNO, High flow nasal cannula oxygen therapy; ICU, Intensive care unit; IMV, Invasive mechanical ventilation; LDH, Lactate dehydrogenase; LOS, Length of hospital stay; NA, not applicable; NIMV, Non-invasive mechanical ventilation; PCT, Procalcitonin; PLT, Platelets; PMNL, Polymorphonuclear leukocytes; RRT, Renal replacement therapy; SOFA, Sequential organ failure score; T.Bil, Total bilirubin; WBC, White blood cell count.

**Figure 2:**
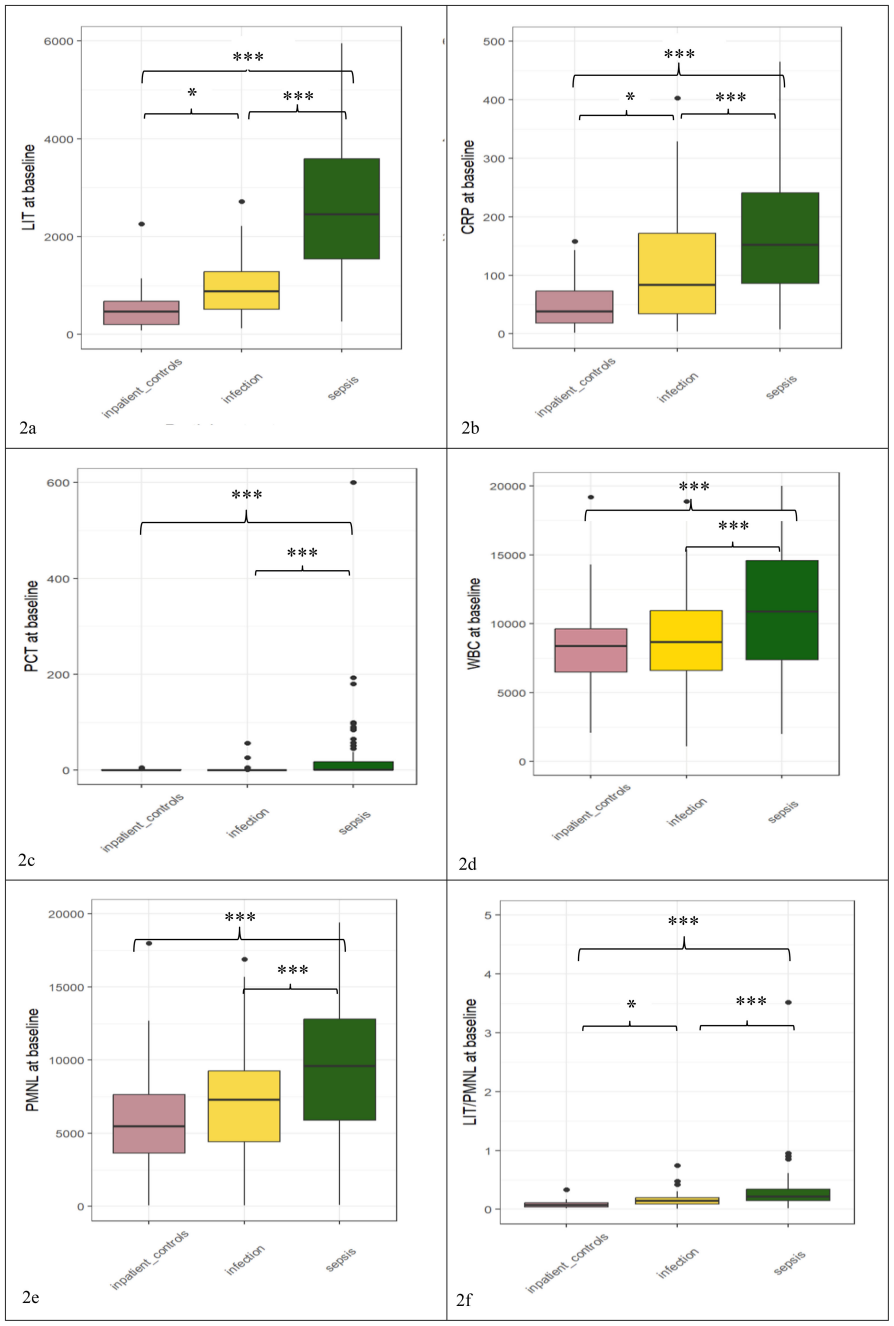
Boxplot of the distribution of inflammatory markers at baseline per participant groups, statistical significance was assessed using Kruskal–Wallis test with Benjamini–Hochberg–adjusted *post hoc* comparisons; p < 0.05 (*), p < 0.01 (**), p < 0.001 (***). **(a)** Distribution of LIT results at baseline, **(b)** Distribution of CRP at baseline, **(c)** Distribution of PCT at baseline, **(d)** Distribution of WBC at baseline, **(e)** Distribution of PMNL at baseline, **(f)** Distribution of LIT/PMNL ratio at baseline, (CRP, C-reactive protein; LIT, Leukocyte ImmunoTest; LIT/PMNL, Leukocyte ImmunoTest-to- polymorphonuclear leukocytes ratio; PCT, Procalcitonin; PMNL, Polymorphonuclear leukocytes; WBC, White blood cell count).

Baseline LIT values demonstrated a progressive increase across the three diagnostic groups, with median values rising from 470 (IQR: 204–622) in inpatient controls to 882 (498–1337) in patients with infection and 2466 (1500–3854) in those with sepsis (adjusted p<0.05 for all comparisons) (**Table 2**, **Figure 2**). A similar stepwise elevation was observed for CRP levels and LIT/PMNL ratio. PCT levels remained comparable between inpatient controls and infection, but showed a significant increase in sepsis. Although WBC and PMNL counts were also elevated in the sepsis group, no significant differences were detected between inpatient controls and infection (**Table 2**). Importantly, in the initial subgroup analysis conducted before *post hoc* group assignment, baseline LIT values significantly differed across the five study groups, with values increasing in association with the presence of infection and the subsequent development of sepsis and septic shock (**Supplementary Table 1**, **Supplementary Figure 1**).

**Table 2:** Baseline inflammatory markers in the three study groups (inpatient control, infection, and sepsis).

Inflammatory markers	Inpatient control (n=29)	Infection (n=47)	Sepsis (n=106)	P value
LIT (RLU)*	470 (204-622)^a,b^	882 (498-1337)^a,c^	2466 (1500-3854)^b,c^	**<0.001**
CRP (mg/L)*	36 (11-70.1)^a,b^	50.5 (14.5-134)^a,c^	156.5 (87.2-242)^b,c^	**<0.001**
PCT (ng/mL)*	0.12 (0.06-0.62)^b^	0.2 (0.075-0.58)^c^	2.02 (0.72-20.4)^b,c^	**<0.001**
WBC (/µL)*	8100 (6400-9800)^b^	8500 (5800-10300)^c^	13250 (8800-19000)^b,c^	**<0.001**
PMNL (/µL)*	5200 (3300-7700)^b^	6800 (4300-8900)^c^	11100 (8000-16800)^b,c^	**<0.001**
LIT/PMNL*	0.09 (0.04-0.11)^a,b^	0.14 (0.08-0.21)^a,c^	0.22 (0.15-0.35)^b,c^	**<0.001**

*median (Q1-Q3), p-values refer to Kruskal-Wallis test across all three groups. Bold values indicate statistical significance (p < 0.05).

Superscript letters indicate significant differences in pairwise comparisons after Benjamini–Hochberg correction: groups with different superscript letters differ significantly (adjusted p < 0.05).

**^a^** inpatient control vs infection.

**^b^** inpatient control vs sepsis.

**^c^** infection vs sepsis.

CRP, C-reactive protein; LIT, Leukocyte ImmunoTest; LIT/PMNL, Leukocyte ImmunoTest-to-polymorphonuclear leukocytes ratio; PCT, Procalcitonin; PMNL, Polymorphonuclear leukocytes; WBC, White blood cell count.

### 3.2 Correlation of LIT with conventional inflammatory markers

**Figure 3:**
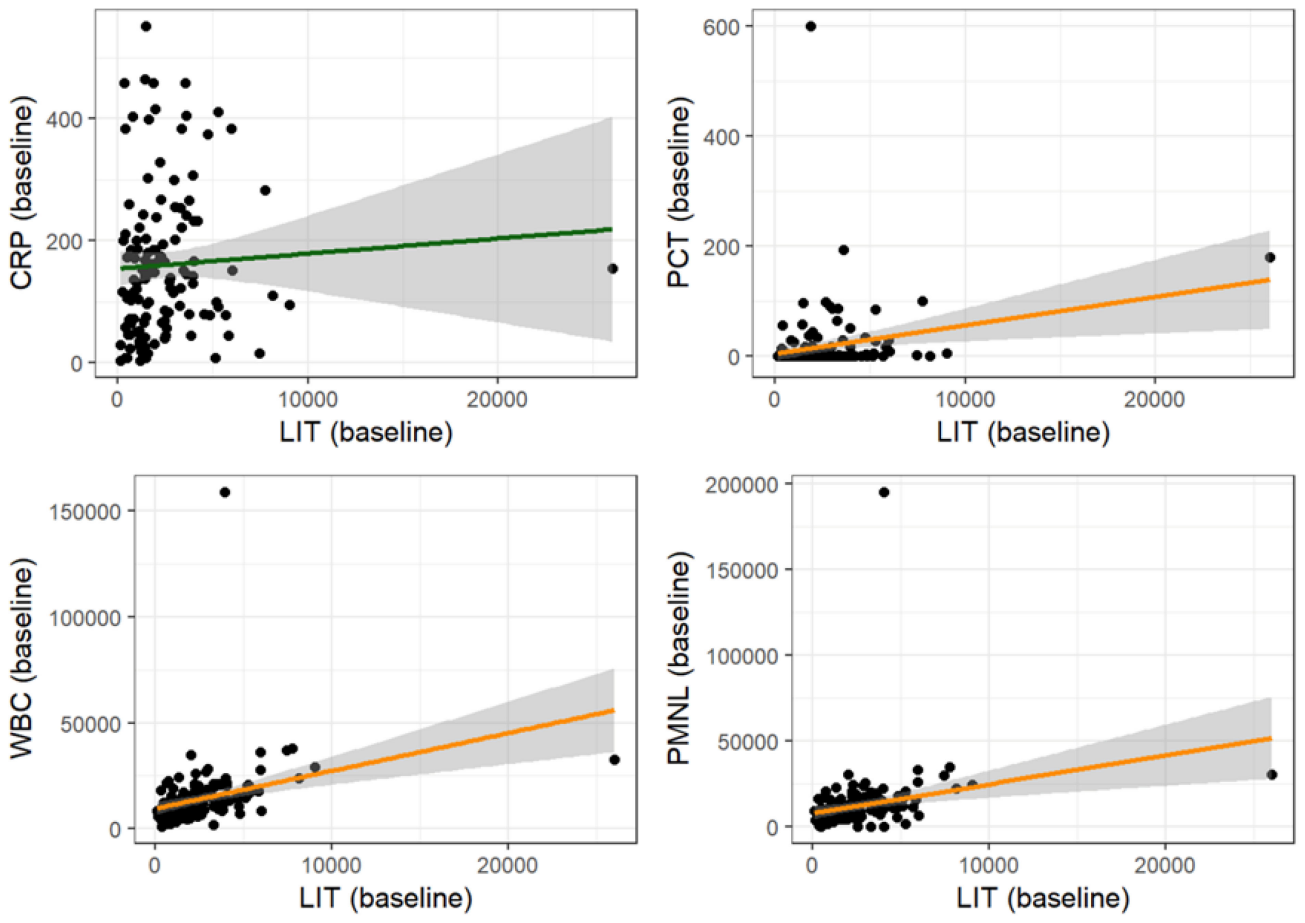
Scatter plots of the baseline LIT results and other inflammatory markers across the overall cohort (n=265).

Spearman’s correlation analysis was conducted to assess the relationship between LIT and other inflammatory parameters across the overall cohort (n=265) (**Figure 3**). In the overall population, LIT showed no meaningful correlation with CRP (rho = 0.189, p = 0.034) or PCT (rho = 0.283, p = 0.001). Correlation patterns were also explored within diagnostic subgroups (inpatient controls, infection, and sepsis), and detailed results are presented in **Supplementary Table 3**.

### 3.3 Impact of diabetes and malignancy status on baseline LIT values in infection, sepsis, and septic shock

Two-way ANOVA models were used to examine the effects of diagnostic group (inpatient controls, infection, and sepsis) and chronic inflammatory conditions (diabetes mellitus and malignancy) on baseline LIT values, as well as potential interaction effects between these factors. In both models, the diagnostic group showed a significant main effect on LIT values (diabetes model: F(2,176) = 19.62, p < 0.001; malignancy model: F(2,176) = 10.69, p < 0.001), indicating that LIT differed meaningfully across clinical categories. However, neither diabetes (F(1,176) = 0.002, p = 0.966) nor malignancy (F(1,176) = 0.046, p = 0.830) demonstrated significant main effects on LIT, and no significant group × diabetes (F(2,176) = 0.086, p = 0.918) or group × malignancy (F(2,176) = 0.070, p = 0.933) interaction was observed (**Supplementary Figure 2**).

### 3.4 Diagnostic performance of inflammatory markers for infection and sepsis

**Figure 4:**
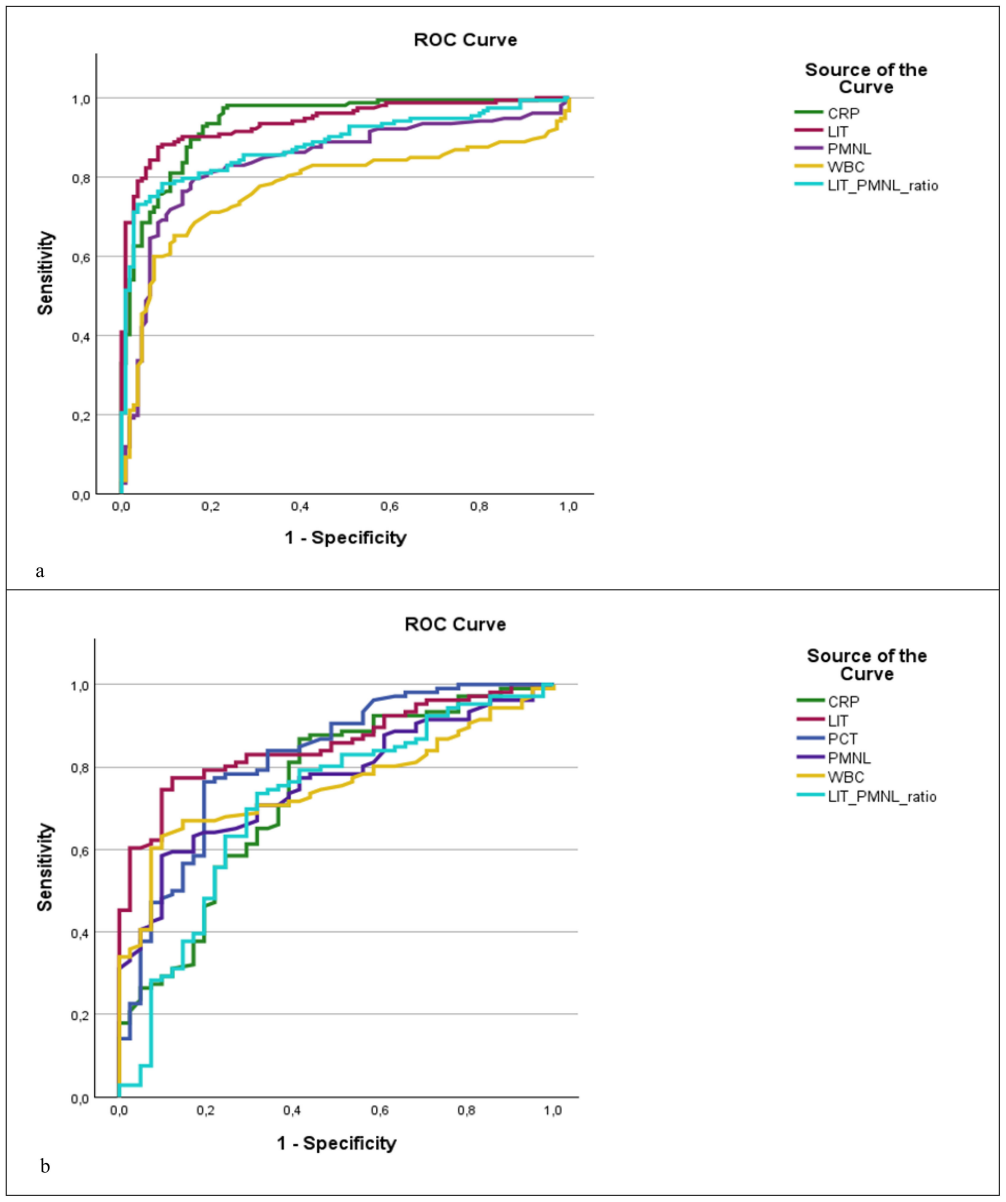
Comparison of inflammatory markers for diagnosis of infection and sepsis with ROC curve analysis. For the diagnosis of infection, the AUC values were 0.939 (95% CI: 0.911–0.968) for LIT, 0.937 (95% CI: 0.908–0.967) for CRP, 0.883 (95% CI: 0.841–0.924) for the LIT/PMNL ratio, 0.841 (95% CI: 0.789–0.892) for PMNL, and 0.744 (95% CI: 0.715–0.833) for WBC. For the diagnosis of sepsis, the AUC values were 0.855 (95% CI: 0.795–0.915) for LIT, 0.820 (95% CI: 0.743–0.898) for PCT, 0.800 (95% CI: 0.687–0.841) for PMNL, 0.739 (95% CI: 0.647–0.832) for CRP, 0.713 (95% CI: 0.617–0.810) for the LIT/PMNL ratio, and 0.700 (95% CI: 0.678–0.832) for WBC. *PCT was not included in the ROC analysis for infection diagnosis due to substantial missing data among outpatient controls, where measurements were available only in a limited subset based on clinical indication. (**(a)** ROC curves for diagnosis of infection, **(b)** ROC curves for diagnosis of sepsis AUC, Area under the curve; CRP, C-reactive protein; LIT, Leukocyte ImmunoTest; LIT/PMNL, Leukocyte ImmunoTest-to-polymorphonuclear leukocytes ratio; PCT, procalcitonin; PMNL, Polymorphonuclear leukocytes; WBC, White blood cell count).

ROC curve analyses were performed separately for infection and sepsis to evaluate the diagnostic accuracy of inflammatory biomarkers (**Figure 4**). LIT yielded an AUC of 0.939 (95% CI: 0.911–0.968) in the diagnosis of infection, which was numerically the highest among the evaluated markers. The corresponding sensitivity and specificity were 90.8% and 78.2%, respectively, at an optimal cut-off of 418.5. CRP and the LIT/PMNL ratio followed closely with AUCs of 0.937 (95% CI: 0.908–0.967) and 0.883 (95% CI: 0.841–0.924), respectively, while PMNL [AUC: 0.841 (95% CI: 0.789–0.892)] and WBC [AUC: 0.744 (95% CI: 0.715–0.833)] demonstrated comparatively lower discriminatory capacity. Pairwise comparisons confirmed that LIT was significantly superior to WBC (p < 0.0001) and PMNL (p = 0.0011), whereas differences with CRP did not reach statistical significance (**Supplementary Table 5**).

For the diagnosis of sepsis, LIT showed numerically the highest AUC among the evaluated markers [0.855 (95% CI: 0.795–0.915)], with 83.0% sensitivity and 68.3% specificity at a cut-off of 1200. PCT [AUC: 0.820 (95% CI: 0.743–0.898)], PMNL [AUC: 0.800 (95% CI: 0.687–0.841)], and CRP [AUC: 0.739 (95% CI: 0.647–0.832)] showed comparable performance, while WBC [AUC: 0.700 (95% CI: 0.678–0.832)] and the LIT/PMNL ratio [AUC: 0.713 (95% CI: 0.617–0.810)] had lower AUCs. Compared to LIT, statistically significant differences were observed with CRP (p = 0.039), WBC (p = 0.048), and the LIT/PMNL ratio (p = 0.021), while differences with PCT and PMNL were not significant.

### 3.5 Association between LIT trajectories and survival outcomes across patient subgroups

**Figure 5:**
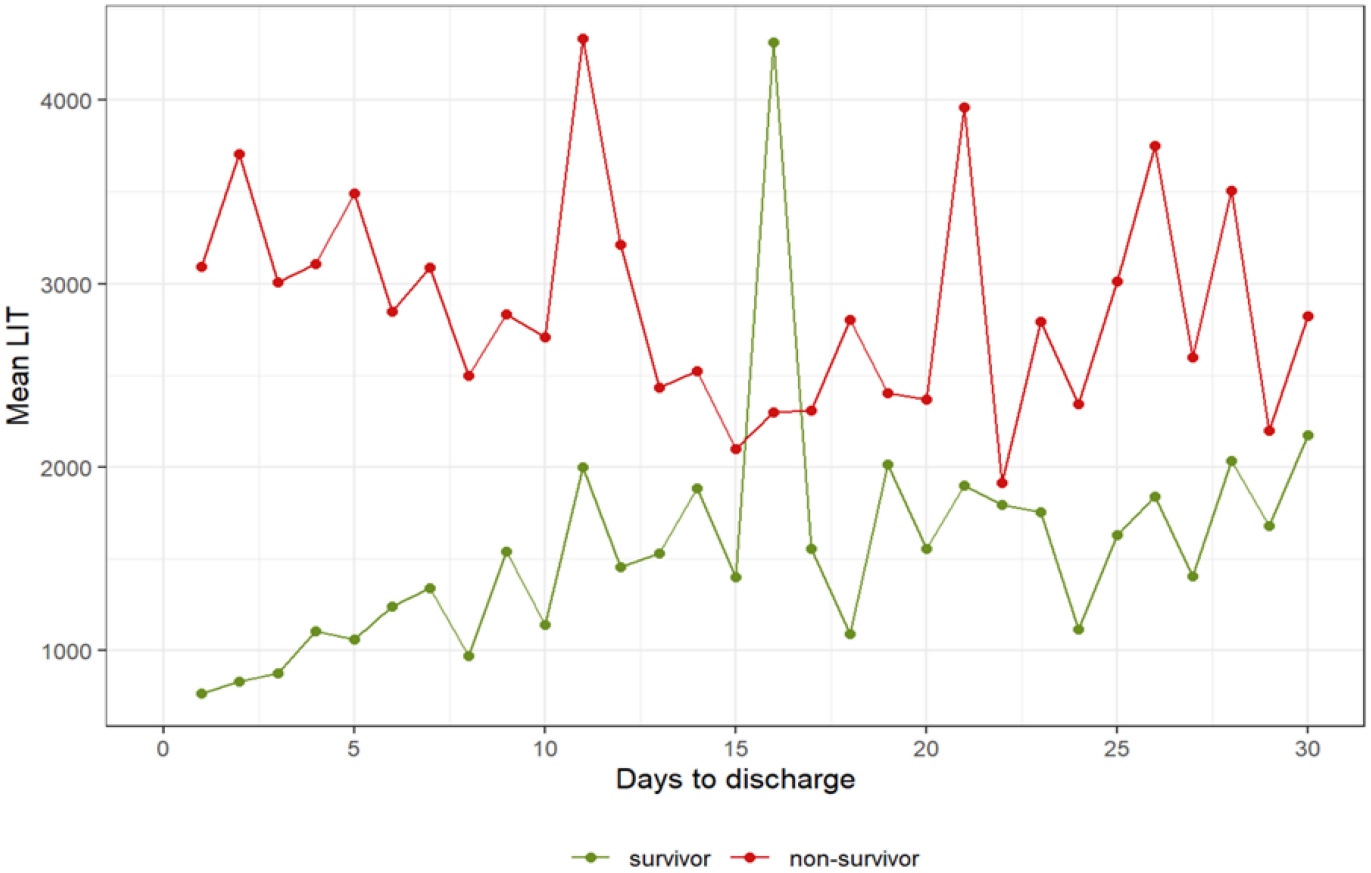
Trajectories of average LIT per number of days from discharge from hospital for survivor and non-survivor all patients (up to 30 days length of stay).

Graphical comparisons of LIT trajectories were generated based on patient status among those with infection, sepsis, and septic shock, according to survival outcomes at hospital discharge, up to 30 days from admission. As illustrated in **Figure 5**, survivors demonstrated a decline in LIT values over time, whereas non-survivors maintained elevated LIT values throughout the ICU stay, this divergence was more pronounced in the sepsis and septic shock subgroups (**Supplementary Figures 3**-**5**).

A joint model combining linear mixed-effects and survival analysis was performed to evaluate the association between LIT values and mortality. An increase in LIT was significantly associated with higher mortality risk, with a hazard ratio (HR) of 1.601 (95% CI: 1.153–2.223; p = 0.005). When evaluated by clinical subgroup, sepsis (HR: 2.836, 95% CI: 1.019–7.895; p = 0.046) and septic shock (HR: 2.751, 95% CI: 1.019–7.895; p = 0.048) were significantly associated with increased mortality compared to inpatient controls, whereas infection was not (HR: 1.486, 95% CI: 0.436–5.064; p = 0.526).

## 4 Discussion

In this study, we evaluated the diagnostic and prognostic utility of the LIT, a rapid assay that quantifies neutrophil-derived ROS production, across a broad spectrum of patients ranging from healthy controls to those with septic shock. Our findings demonstrated a progressive elevation in LIT values corresponding to the increasing severity of infection, with the highest levels observed in patients with sepsis. Correlation analyses revealed only weak associations between LIT and conventional inflammatory markers, suggesting that LIT may capture distinct dimensions of the host immune response. Among conventional inflammatory markers, LIT demonstrated good performance in ROC analyses, with AUC values comparable to CRP for identifying infection and to PCT for identifying sepsis. Furthermore, the persistent elevation of LIT results among non-survivors, particularly those with sepsis and septic shock, suggests a potential association with adverse outcomes. Collectively, these findings suggest that LIT may serve as a neutrophil-based biomarker with potential diagnostic and prognostic relevance in the management of infectious diseases, especially in sepsis.

Neutrophils are a pivotal component of the innate immune system and play a central role in host defense against bacterial, fungal, and viral infections (3, 7, 28, 29). Once activated, they migrate rapidly to infection sites and execute key effector functions, such as phagocytosis, degranulation, and the production of ROS. During the COVID-19 pandemic, elevated neutrophil-to-lymphocyte ratios (NLR) were associated with worse outcomes, highlighting the significance of neutrophil activation and dysregulation in severe infections (30, 31). Excessive ROS production and the formation of NETs have since been recognized not only as markers of infection severity but also as contributors to tissue damage and immune dysregulation in critical illness (8, 11, 29, 32, 33). Given this background, there is increasing interest in bedside assays that can quantify neutrophil function in a dynamic manner, particularly ROS activity. The LIT, which measures neutrophil-derived ROS from whole blood within minutes, has emerged as a practical tool to capture this dynamic immune response at the point of care (20). Recent studies conducted during the COVID-19 pandemic further underscored the relevance of dynamic neutrophil function assessment (23, 24). In particular, LIT values were elevated in patients with viral infections compared to controls, and persistently high in those who developed nosocomial infections, suggesting a potential role for neutrophil-derived ROS in sepsis (23). In line with these observations, our study demonstrated significantly elevated LIT values in patients with infection and sepsis compared to both inpatient controls. Unlike prior studies limited to viral infections, we included a broader clinical spectrum and confirmed that LIT values increase with infection severity. Moreover, the distinction observed between inpatient controls and infected patients highlights the sensitivity of this test in detecting neutrophil activation beyond overt clinical signs of sepsis. From this perspective, it is essential to recognize that current sepsis definitions rely primarily on downstream indicators of organ dysfunction, such as those reflected in the SOFA criteria, rather than upstream markers of immune activation (1, 3, 25). This conceptual gap underscores the potential utility of the LIT assay, which captures a dynamic functional neutrophil response through real-time measurement of ROS production. As ROS generation is not only an early feature of innate immune activation but also mechanistically linked to later organ damage, LIT may represent a promising biomarker that could help identify sepsis at an earlier, immunologically active phase.

LIT demonstrated a diagnostic profile comparable to that of CRP and PCT in detecting infection and sepsis, with numerically higher AUCs and sensitivity-specificity values. These findings align with prior reports indicating that PCT generally exhibits higher specificity than CRP in sepsis diagnosis, with pooled sensitivities ranging from 77–85% and specificities from 75–83% (34). In contrast, CRP’s specificity has been reported as low as 60–61%, despite comparable sensitivity (34). Furthermore, for the diagnosis of infection, meta-analyses have shown that PCT retains higher diagnostic accuracy than CRP, with sensitivity/specificity approximating 88%/81% for PCT and 75%/67% for CRP (35, 36). It should be noted, however, that PCT is a widely used biomarker in the diagnosis of bacterial sepsis, and, similar to CRP, a range of cut-off values has been reported in the literature, resulting in variable sensitivity and specificity estimates (37). LIT provides a dynamic and functional readout of innate immune activation by capturing neutrophil-derived ROS activity. Conventional biomarkers such as CRP and PCT, which reflect downstream hepatic or neuroendocrine responses, exhibit significant delays in their kinetics. CRP typically peaks within 2–3 days and has a half-life of approximately 19 hours, while PCT peaks at 36–50 hours with a similar half-life (2, 38). LIT may provide a more dynamic reflection of host–pathogen interaction. These characteristics may provide a potential advantage for use in the early phase, particularly in clinical settings where risk stratification is critical. However, in our study, pairwise AUC comparisons using the DeLong test revealed no significant difference between LIT and CRP for infection diagnosis, while in sepsis diagnosis, LIT showed a significant difference compared with CRP but not with PCT. This finding suggests the need for larger, prospectively designed cohorts to validate these observations. It should also be noted that CRP and PCT reflect different biological pathways, and therefore direct comparisons with LIT should be interpreted within the context of these mechanistic differences. While early studies in predominantly surgical populations suggested that PSP may outperform conventional biomarkers in distinguishing infection and sepsis, its performance in nosocomial infections appears to be more comparable to established markers (39, 40). Unlike parts of the PSP literature that specifically address diagnostic performance in the presence of non-infectious SIRS, the ability of LIT to discriminate infection in such contexts was not directly evaluated in our study (12, 18, 39–42). This is partly related to the composition of our control group, which mainly consisted of patients hospitalized for non-infectious medical conditions, with a relatively limited number of patients undergoing major surgical interventions. Therefore, further studies are needed to clarify the performance of LIT in differentiating infection from non-infectious inflammatory states, particularly in high-risk surgical and critically ill populations. Moreover, as the study was exploratory in nature, we did not perform a prior power analysis to determine the required sample size to detect differences in diagnostic performance. This should be considered as a methodological limitation.

The prognostic utility of LIT in our cohort was best reflected through its longitudinal trajectory. Persistently elevated LIT values during the ICU stay, particularly among non-survivors with septic shock, suggest that the dynamic pattern of neutrophil-derived ROS production may provide a more informative signal of clinical outcomes than static, single time-point measurements. This finding is noteworthy because previous studies have highlighted the limited prognostic accuracy of conventional biomarkers such as CRP and PCT in sepsis. CRP, an acute-phase reactant produced by the liver, lacks specificity for sepsis and may not reliably reflect the degree of systemic inflammation, thereby limiting its prognostic value (43, 44). Similarly, although PCT is widely used to guide antibiotic therapy, its levels vary depending on the infectious etiology and are influenced by the timing and nature of the initial insult, which may limit its reliability as a stand-alone prognostic marker (45–48). Our previous study in patients with severe COVID-19 further supports the prognostic relevance of LIT, demonstrating that elevated values were associated with poor outcomes irrespective of microbial origin (23). These findings suggest that LIT may have potential as a prognostic biomarker in critical care. Moreover, recent studies linking excessive ROS production and NETs formation to tissue damage and organ dysfunction provide a mechanistic rationale for the association between sustained high LIT values and poor clinical outcomes (32, 33).

Finally, in conditions such as diabetes and malignancy, neutrophils are known to exhibit both impaired functional capacity and heightened pro-inflammatory activity (49–51). These alterations may influence host immune responses, contribute to an increased vulnerability to infection, and potentially affect baseline LIT values. However, in our cohort, baseline LIT values did not significantly differ according to diabetes and malignancy status. Unlike Li et al., who observed elevated LIT scores in malignancies, we found no such association (51). This discrepancy may be due to the dominant immune activation caused by the concurrent infection, which could mask cancer-related immune alterations that might otherwise be detectable by LIT under non-infectious conditions. Another possible explanation is the fact that most malignancies in our cohort were either under active treatment or in remission at the time of LIT measurement, which may have attenuated their influence on the measured immune response. These findings suggest that while LIT effectively differentiates infection severity, its performance appears unaffected by the presence of diabetes or cancer in this study population.

### 4.1 Strengths and limitations

This study has several notable strengths. First, it is the first to evaluate the diagnostic and prognostic utility of LIT across a broad clinical spectrum, from healthy individuals to critically ill patients with septic shock, including those in ICU settings. Beyond simply assessing LIT in infection and sepsis, our analysis also examined its performance in the context of chronic inflammatory conditions such as diabetes and malignancy, providing insights into potential modifiers of baseline LIT values. Second, the study directly compared LIT with widely used conventional inflammatory biomarkers, including CRP and PCT, allowing assessment of its relative performance in a clinical context. Unlike many experimental biomarkers that have shown promise in research settings but failed to translate into practice, LIT is rapid (~15 minutes), and does not require complex preprocessing, making it suitable for daily laboratory workflows. Finally, the test’s ability to capture dynamic immune activity through ROS production, together with potential diagnostic and prognostic relevance and mechanistic basis, suggests translational applicability for infection management in critically ill populations.

The primary limitation of this study is the lack of a predefined power analysis, which was not feasible given that this was the first investigation to evaluate LIT in the context of sepsis. However, retrospective calculations based on previously reported LIT values between COVID-19 patients and healthy controls suggest that the study may have had sufficient statistical power to detect clinically meaningful differences. Another potential limitation is the composition of the control group for infection diagnosis, which included both healthy volunteers and inpatient controls. While this heterogeneity might be viewed as a methodological concern, it may also enhance the real-world applicability of the findings, especially in the absence of established LIT thresholds. The study population was limited to ICU and internal medicine ward patients, without including emergency department admissions, where early recognition of sepsis is particularly critical. Operational limitations primarily drove this design constraint. While this limits the generalizability of our findings to early triage settings, the study nonetheless provides a biologically relevant proof-of-concept supporting the utility of LIT in detecting and stratifying infection and sepsis severity among critically ill and hospitalized patients. Given its rapid and functional nature, LIT may also have potential applicability in emergency department or very early clinical settings; however, such use remains to be prospectively validated. In addition, patients who died within the first 24 hours were excluded, as LIT measurements and complete diagnostic evaluation could not be reliably performed in this time frame, and their inclusion could have introduced substantial misclassification bias due to undefined exposure (LIT values) and uncertain diagnostic categorization. Moreover, data on the use of corticosteroids or other immunosuppressive therapies, which may influence neutrophil responses, were not available, potentially confounding the interpretation of neutrophil activity. Finally, as a single-center observational study, our findings require external validation before clinical implementation.

### 4.2 Future directions

Future studies should focus on validating the diagnostic and prognostic performance of LIT in multicenter, prospective cohorts, ideally with diverse patient populations and etiologies of sepsis. In addition, efforts should be directed toward defining optimal cutoff values for LIT in clinical practice and exploring its potential integration into sepsis screening algorithms. Longitudinal studies assessing LIT, particularly in relation to interventions aimed at improving patient outcomes, such as the timing of antibiotic therapy, immunomodulatory therapies, or ICU triage, could further elucidate its clinical utility. Moreover, mechanistic investigations into the relationship between neutrophil ROS activity, NET formation, and organ dysfunction may provide deeper insights.

## 5 Conclusion

In conclusion, this study is one of the first real-world evaluations of a functional immune assay that reflects neutrophil-derived ROS in the clinical setting of infection and sepsis. By capturing the dynamic nature of neutrophil-derived ROS activity, LIT provides a dynamic and functionally relevant measure of immune activation, distinguishing it from conventional inflammatory biomarkers not only by its real-time functional readout but also by reflecting a potentially distinct immunological pathway of innate immune cell activity. Its rapid, bedside applicability, combined with its ability to reflect both infection severity and clinical outcomes, suggests that LIT may represent a promising candidate for potential integration into routine sepsis assessment protocols. Future prospective multicenter studies are warranted to validate these findings and to further evaluate the clinical utility of LIT across diverse critical care settings. Additionally, it remains to be investigated whether LIT-guided strategies can enhance diagnostic accuracy, improve risk stratification, track immune recovery over time, and ultimately contribute to better patient outcomes in managing sepsis.

## Data Availability

The datasets generated and analyzed during the current study are not publicly available due to planned *post hoc* analyses as part of an ongoing research extension. Requests to access the datasets should be directed to nazlihan@gazi.edu.tr.
