# Supplementary material for: Neutrophil-derived ROS as a rapid functional biomarker: diagnostic and prognostic performance of the Leukocyte ImmunoTest in infection and sepsis
Source: Front Immunol. 2026 Jun 29;17:1829226. doi: 10.3389/fimmu.2026.1829226 (PMC13357141; doi:10.3389/fimmu.2026.1829226)

Supplementary Material

# Supplemental Methods – Statistical Analyses

Continuous variables were examined for normality using the Shapiro–Wilk test and visual inspection of histograms and Q-Q plots. Data are presented as means ± standard deviation (SD) for normally distributed variables, or medians with interquartile ranges (Q1–Q3) for non-normally distributed variables. Categorical variables are expressed as frequencies and percentages. Between-group comparisons of continuous variables were performed using the Kruskal–Wallis test for non-parametric data, followed by Dunn’s post-hoc pairwise comparisons when applicable. For categorical variables, Chi-squared tests were applied, or Fisher’s exact test in cases of sparse data. To control for type I error due to multiple comparisons, the Benjamini–Hochberg false discovery rate (FDR) correction was applied to all p-values, with a significance threshold set at 0.05.

The diagnostic performance of Leukocyte ImmunoTest (LIT) and conventional inflammatory biomarkers [e.g., C-reactive protein (CRP), Procalcitonin (PCT)] was assessed using receiver operating characteristic (ROC) curve analysis for infection and sepsis diagnosis in the relevant study populations. PCT was not included in ROC analyses for infection diagnosis due to substantial missing data among outpatient controls, as PCT measurements were available only in a limited subset of this group based on clinical indication. In addition, given that the study population included both bacterial and viral infections, the diagnostic performance of PCT for infection diagnosis was considered less representative in this broader context. Areas under the curve (AUCs) with 95% confidence intervals (CI) were calculated, and differences between AUCs were tested using the DeLong method. Optimal cut-off values were determined using the Youden index, with corresponding sensitivity, specificity, positive predictive value (PPV), and negative predictive value (NPV) calculated.

Correlation analyses between LIT and other inflammatory markers were conducted using Spearman’s rank correlation coefficient (ρ), with interpretation based on standard effect size thresholds (|ρ| = 0.1–0.3 small, 0.3–0.5 moderate, >0.5 strong).

Survival analysis was performed by stratifying patients into survivors and non-survivors at ICU or hospital discharge. To explore longitudinal associations between repeated LIT measurements and survival, a joint modeling framework was used, combining a Cox proportional hazards model with a linear mixed-effects submodel. LIT values, which exhibited right-skewed distribution, were log-transformed before modeling. The longitudinal submodel included time since admission, clinical condition, and time-by-condition interaction as fixed effects, while random intercepts and slopes accounted for within-patient variability. The survival submodel incorporated baseline clinical condition as a time-independent covariate, with time-varying LIT trajectories as dynamic predictors of mortality risk. In addition, clinically relevant covariates including age, sex, comorbidities, and length of stay were explored during model development. These variables were not retained in the final model, as they were not significantly associated with the outcome and did not improve model fit, as assessed by information criteria. Length of stay was retained in selected models due to its clinical relevance. Model selection was guided by a preference for parsimony while preserving clinically meaningful variables.

All analyses were performed in R (version 4.4.1) using tidyverse for data manipulation and visualization, lme4 and lmerTest for mixed-effects modeling, survival and JM packages for joint models, pROC for ROC analysis, and data.table for efficient data handling. IBM SPSS Statistics (version 22) was additionally used for descriptive statistics, Kruskal–Wallis tests, and pairwise post-hoc comparisons. The statistical workflow was developed in close collaboration with a biostatistician team to ensure reproducibility, transparency, and adherence to modern standards for biomedical data analysis.

# Supplementary Results

## Five groups comparisons, before post hoc group assigned

The baseline distribution of six inflammatory markers across the study groups is presented in **Supplemental Figure 1.** and **Supplemental Table 1**. Compared to the control groups, levels of LIT, CRP, PCT, WBC, PMNL, and the LIT/PMNL ratio increased with the presence and severity of infection. Only LIT showed significant differences among these markers across all pairwise group comparisons. CRP, PCT, WBC, and PMNL did not significantly differentiate infection from inpatient controls, and no significant differences were observed between the sepsis and septic shock groups for any marker other than LIT. Adjusted p-values for all comparisons are reported in **Supplemental Table 2**.


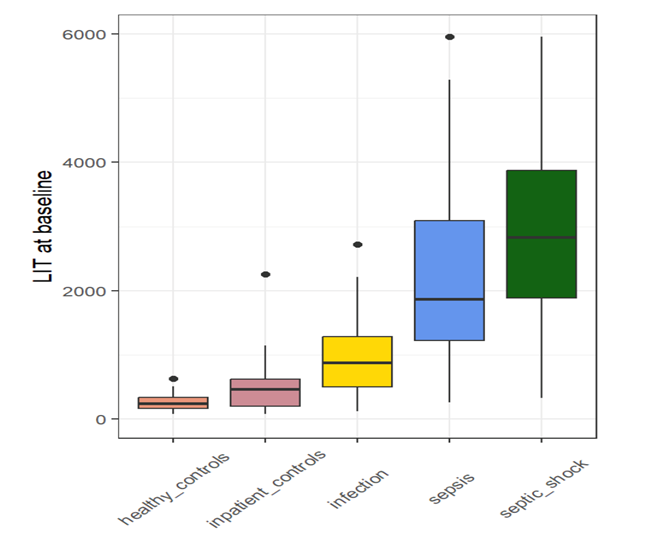


**Supplemental Figure 1**. Boxplot and Dot plot (mean in red) of the distribution of LIT results at baseline per participant group

| **Supplemental Table 1.** Summary statistics of inflammatory markers at baseline by five groups of patients | | | | | |
| --- | --- | --- | --- | --- | --- |
|  | **Healthy control**  **(n=83)** | **Inpatient control**  **(n=29)** | **Infection**  **(n=47)** | **Sepsis**  **(n=49)** | **Septic shock**  **(n=57)** |
| **LIT*** | 241 (169.5-339.5) | 470 (204-681) | 882 (517-1281) | 1876 (1298-3273) | 3271 (2252-4017) |
| **CRP*** | 2.28 (1.75-3.35) | 36.0 (11.0-70.1) | 50.5 (14.5-134.0) | 166.0 (86.6-227.0) | 143.0 (91.0-244) |
| **PCT*** | 0.02 (0.02-0.02) | 0.12 (0.06-0.62) | 0.20 (0.08-0.58) | 1.08 (0.385-12.86) | 5.08 (0.91-21.0) |
| **WBC*** | 6300 (5600-7500) | 8100 (6400-9800) | 8500 (5800-10300) | 12400 (8600-17700) | 13400 (10200-20400) |
| **PMNL*** | 3500 (3100-4700) | 5200 (3300-7700) | 6800 (4300-8900) | 11000 (7500-15000) | 11500 (8300-18700) |
| **LIT/PMNL*** | 0.06 (0.05-0.09) | 0.09 (0.04-0.11) | 0.14 (0.08-0.21) | 0.18 (0.13-0.29) | 0.25 (0.19-0.36) |
| *median (Q1-Q3)  **CRP**; C-reactive protein, **LIT;** Leukocyte ImmunoTest, **LIT/PMNL**; Leukocyte ImmunoTest-to-polymorphonuclear leukocytes ratio, **PCT**; Procalcitonin, **PMNL**; Polymorphonuclear leukocytes, **WBC**; White blood cell count | | | | | |

## Supplemental Table 2. Adjusted p values of pairwise comparisons of inflammatory markers at baseline by five groups of patients

| **Supplemental Table 2.** Adjusted p values of pairwise comparisons of inflammatory markers at baseline by five groups of patients | | | | | | | |
| --- | --- | --- | --- | --- | --- | --- | --- |
| **Groups of Pairwise Comparison** | | **LIT** | **CRP** | **PCT** | **WBC** | **PMNL** | **LIT/PMNL** |
| Healthy control | Inpatient control | **0.022** | **0.002** | **0.03** | **0.006** | **0.005** | 0.197 |
| Healthy control | Infection | **0.000** | **0.003** | **0.01** | **0.019** | **0.002** | **0.003** |
| Healthy control | Sepsis | **0.000** | **0.005** | **0.003** | **0.005** | **0.005** | **0.005** |
| Healthy control | Septic Shock | **0.000** | **0.001** | **0.003** | **0.001** | **0.01** | **0.000** |
| Inpatient control | Infection | **0.023** | 0.345 | 0.561 | 0.992 | 0.513 | **0.004** |
| Inpatient control | Sepsis | **0.000** | **0.002** | **0.002** | **0.002** | **0.002** | **0.002** |
| Inpatient control | Septic Shock | **0.000** | **0.002** | **0.001** | **0.003** | **0.003** | **0.003** |
| Infection | Sepsis | **0.001** | **0.001** | **0.002** | **0.003** | **0.001** | **0.038** |
| Infection | Septic Shock | **0.000** | **0.001** | **0.005** | **0.003** | **0.003** | **0.002** |
| Sepsis | Septic Shock | **0.048** | 0.933 | 0.128 | 0.497 | 0.462 | 0.1 |
| Dunn’s test-significant comparisons only **p-value<0.05**, p-values have been adjusted for multiple comparisons using the Benjamini-Hochberg method. **CRP**; C-reactive protein, **LIT;** Leukocyte ImmunoTest, **LIT/PMNL**; Leukocyte ImmunoTest-to-polymorphonuclear leukocytes ratio **PCT**; Procalcitonin, **PMNL**; Polymorphonuclear leukocytes, **WBC**; White blood cell count | | | | | | | |

## Supplemental Table 3. Spearman’s correlation coefficient table for inflammatory markers at baseline per overall and subgroups.

| **Supplemental Table 3.** Spearman’s correlation coefficient table for inflammatory markers at baseline per overall and subgroups. | | | | | | | | |
| --- | --- | --- | --- | --- | --- | --- | --- | --- |
|  | **Overall (n=265)** | | **Inpatient control (n=29)** | | **Infection (n=47)** | | **Sepsis (n=106)** | |
| **Markers** | Rho (95%CI) | p-value | Rho (95%CI) | p-value | Rho (95%CI) | p-value | Rho (95%CI) | p-value |
| LIT,CRP | 0.189 (0.014-0.357) | **0.034** | 0.367 (-0.11-0.653) | **0.05** | 0.336 (0.042-0.577) | **0.022** | 0.06 (-0.138-0.254) | 0.539 |
| LIT,PCT | 0.283 (0.116-0.431) | **0.001** | 0.298 (-0.089-0.606) | **0.117** | -0.036 (-0.348-0.284) | 0.825 | 0.063 (-0.135-0.256) | 0.521 |
| LIT,WBC | 0.579 (0.441-0.69) | **0.000** | 0.578 (0.257-0.784) | **0.001** | 0.347 (0.057-0.582) | **0.017** | 0.607 (0.466-0.707) | **0.000** |
| LIT,PMNL | 0.523 (0.382-0.656) | **0.000** | 0.650 (0.362-0.824) | **0.000** | 0.471 (0.204-0.673) | **0.000** | 0.593 (0.449-0.707) | **0.000** |
| LIT,LIT/PMNL | 0.832 (0.79-0.867) | **0.000** | 0.509 (0.164-0.743) | **0.005** | 0.637 (0.421-0.785) | **0.000** | 0.540 (0.384-0.666) | **0.000** |
| CRP,PCT | 0.449 (0.291-0.591) | **0.000** | 0.587 (0.27-0.789) | **0.000** | 0.324 (0.009-0.581) | **0.039** | 0.363 (0.179-0.522) | **0.000** |
| CRP,WBC | 0.178 (0-0.336) | **0.047** | 0.227 (-0.63-0.556) | 0.236 | 0.257 (-0.045-0.516) | 0.085 | -0.072 (-0.265-0.126) | 0.463 |
| CRP,PMNL | 0.176 (-0.004-0.336) | **0.048** | 0.304 (-0.082-0.61) | 0.109 | 0.304 (0.006-0.552) | **0.04** | -0.009 (-0.205-0.188) | 0.930 |
| CRP, LIT/PMNL | -0.025 (-0.213-0.146) | 0.779 | 0.247 (-0.142-0.571) | 0.196 | 0.076 (-0.228-0.366) | 0.617 | 0.081 (-0.117-0.273) | 0.410 |
| PCT,WBC | 0.123 (-0.052-0.294) | 0.166 | 0.228 (-0.162-0.557) | 0.234 | -0.059 (-0.36-0.315) | 0.715 | 0.086 (-0.112-0.278) | 0.381 |
| PCT,PMNL | 0.121 (-0.056-0.281) | 0.175 | 0.325 (-0.59-0.625) | 0.086 | -0.002 (-0.312-0.315) | 0.992 | 0.126 (-0.72-0.315) | 0.197 |
| PCT, LIT/PMNL | 0.144 (-0.025-0.302) | 0.104 | 0.068 (-0.316-0.433) | 0.726 | 0.019 (-0.299-0.33) | 0.907 | -0.025 (-0.22-0.172) | 0.802 |
| WBC,PMNL | 0.992 (0.842-0.975) | **0.000** | 0.969 (0.932-0.986) | **0.000** | 0.843 (0.730-0.912) | 0.000 | 0.973 (0.96-0.982) | **0.000** |
| WBC, LIT/PMNL | -0.199(-0.348-(-0.025)) | **0.016** | -0.33 (-0.628-0.053) | 0.08 | -0.332 (-0.57-(-0.04) | **0.023** | -0.217 (-0.396-(-0.21) | **0.026** |
| **CRP**; C-reactive protein, **LIT;** Leukocyte ImmunoTest, **LIT/PMNL**; Leukocyte ImmunoTest-to-polymorphonuclear leukocytes ratio **PCT**; Procalcitonin, **PMNL**; Polymorphonuclear leukocytes, **WBC**; White blood cell count | | | | | | | | |

## 2.4 Supplemental Figure 2. Estimated marginal means of baseline LIT values across diagnostic groups, stratified by diabetes and malignancy status. (4a; stratified by diabetes status, 4b; stratified by malignanc status)

| 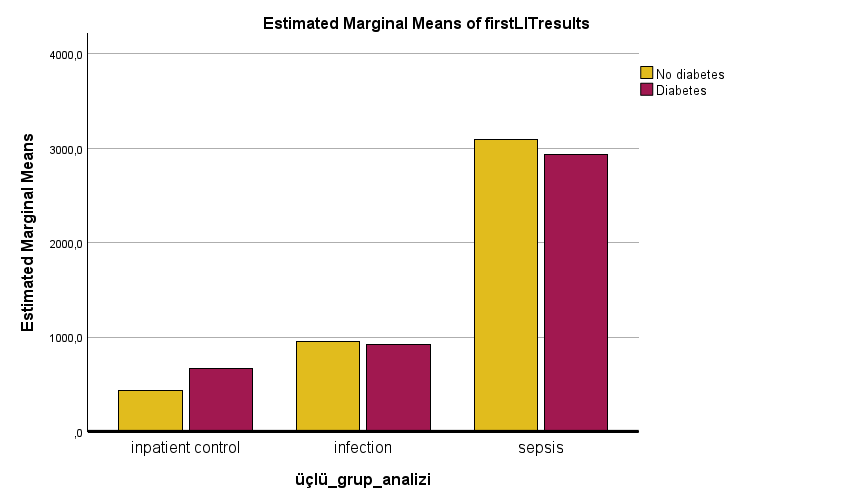  4a |
| --- |
| 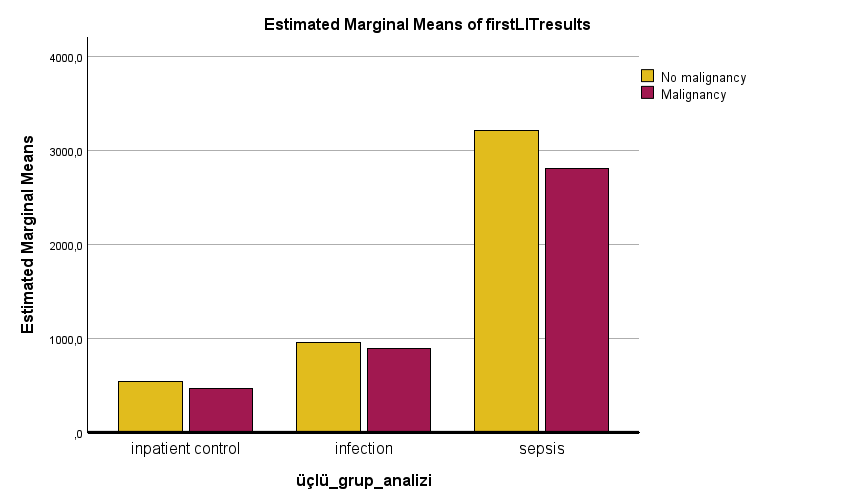  4b |

**Supplemental Figure 2.** Estimated marginal means of baseline LIT values across diagnostic groups, stratified by diabetes and malignancy status. (**4a;** stratified by diabetes status, **4b;** stratified by malignanc status)

## Supplemental Table 4. Comparison of inflammatory markers for diagnosis of infection and sepsis with ROC analysis

| **Supplemental Table 4.** Comparison of inflammatory markers for diagnosis of infection and sepsis with ROC analysis | | | | | | | | | |
| --- | --- | --- | --- | --- | --- | --- | --- | --- | --- |
|  | **Diagnosis of infection*** | | | | **Diagnosis of sepsis**** | | | | |
|  | **AUC** | **Sen (%)** | **Spe (%)** | **Cut-Off** | **AUC** | **Sen (%)** | **Spe (%)** | **Cut-Off** |  |
| **LIT** | 0.939 | 90.8 | 78.2 | 418.5 | 0.855 | 83.0 | 68.3 | 1200 |  |
| **CRP** | 0.937 | 98 | 74.5 | 5.8 | 0.739 | 81.1 | 61.0 | 77.2 |  |
| **PCT***** | - | - | - | - | 0.820 | 84.0 | 65.9 | 0.35 |  |
| **WBC** | 0.774 | 77.6 | 69.1 | 7555 | 0.755 | 70.8 | 65.9 | 10500 |  |
| **PMNL** | 0.841 | 80.3 | 80.9 | 5450 | 0.764 | 73.6 | 61.0 | 8150 |  |
| **LIT/PMNL** | 0.883 | 85.5 | 72.7 | 0.0913 | 0.713 | 76.4 | 61.0 | 0.1513 |  |
| *****within 265 subjects (included outpatient control, inpatient control, infection, sepsis, and septic shock groups)  ******within 153 subjects (included infection, sepsis, and septic shock gropus)  ***PCT was not included in the ROC analysis for infection diagnosis due to substantial missing data among outpatient controls, where measurements were available only in a limited subset based on clinical indication.  **CRP**; C-reactive protein, **LIT;** Leukocyte ImmunoTest, **LIT/PMNL**; Leukocyte ImmunoTest-to-polymorphonuclear leukocytes ratio **PCT**; Procalcitonin, **PMNL**; Polymorphonuclear leukocytes, **Sen**; sensitivity, **Spe**; specificity **WBC**; White blood cell count | | | | | | | | | |

## Supplemental Table 5. Pairwise comparison of AUC values between LIT and other biomarkers for infection and sepsis diagnosis

| **Supplemental Table 5.** Pairwise comparison of AUC values between LIT and other biomarkers for infection and sepsis diagnosis | | | | | | |
| --- | --- | --- | --- | --- | --- | --- |
|  | **Diagnosis of infection*** | | | **Diagnosis of sepsis**** | | |
|  | **ΔAUC** | **Z value** | **P value** | **ΔAUC** | **Z value** | **P value** |
| **LIT vs CRP** | 0.002 | 0.097 | 0.92 | 0.116 | 2.06 | **0.039** |
| **LIT vs PCT***** | - | - | - | 0.035 | 0.7 | 0.48 |
| **LIT vs WBC** | 0.165 | 4.99 | **<0.0001** | 0.100 | 1.98 | **0.048** |
| **LIT vs PMNL** | 0.098 | 3.27 | **0.0011** | 0.091 | 1.73 | 0.083 |
| **LIT vs LIT/PMNL** | 0.056 | 2.00 | **0.045** | 0.142 | 2.30 | **0.021** |
| *****within 265 subjects (included outpatient control, inpatient control, infection, sepsis, and septic shock groups)  ******within 153 subjects (included infection, sepsis, and septic shock gropus)  ***PCT was not included in the ROC analysis for infection diagnosis due to substantial missing data among outpatient controls, where measurements were available only in a limited subset based on clinical indication.  DeLong’s test-significant comparisons only **p-value<0.05**  **ΔAUC;** AUC difference, **CRP**; C-reactive protein, **LIT**; Leukocyte ImmunoTest, **LIT/PMNL**; Leukocyte ImmunoTest-to-polymorphonuclear leukocytes ratio **PCT**; Procalcitonin, **PMNL**; Polymorphonuclear leukocytes, **SE; S**tandart error, **WBC**; White blood cell count | | | | | | |

## Supplemental Figure 3. Trajectories of average LIT per number of days from discharge from hospital for survivor and non-survivor infection patients (up to 30 days length of stay)


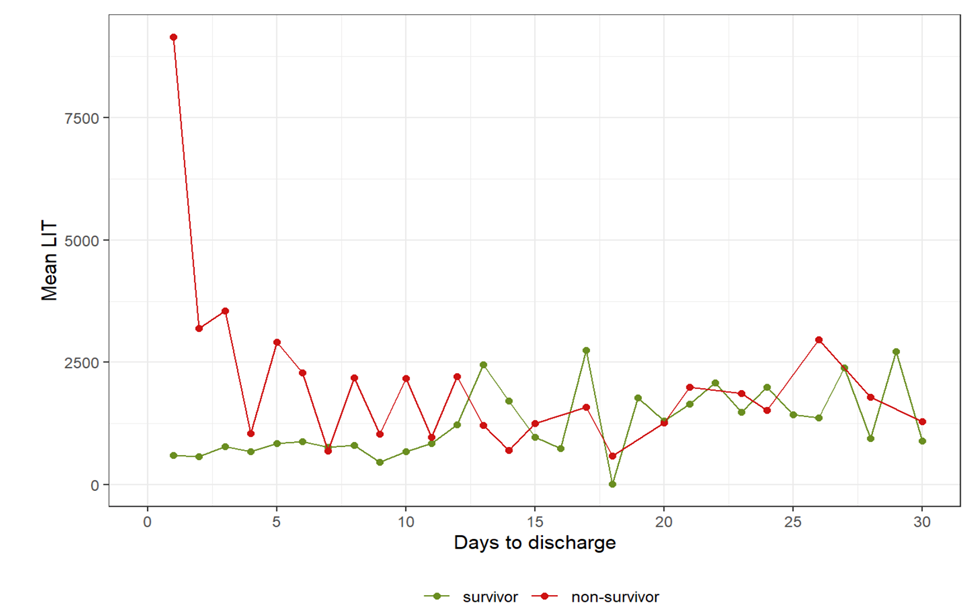


## Supplemental Figure 4. Trajectories of average LIT per number of days from discharge from hospital for survivor and non-survivor sepsis patients (up to 30 days length of stay)


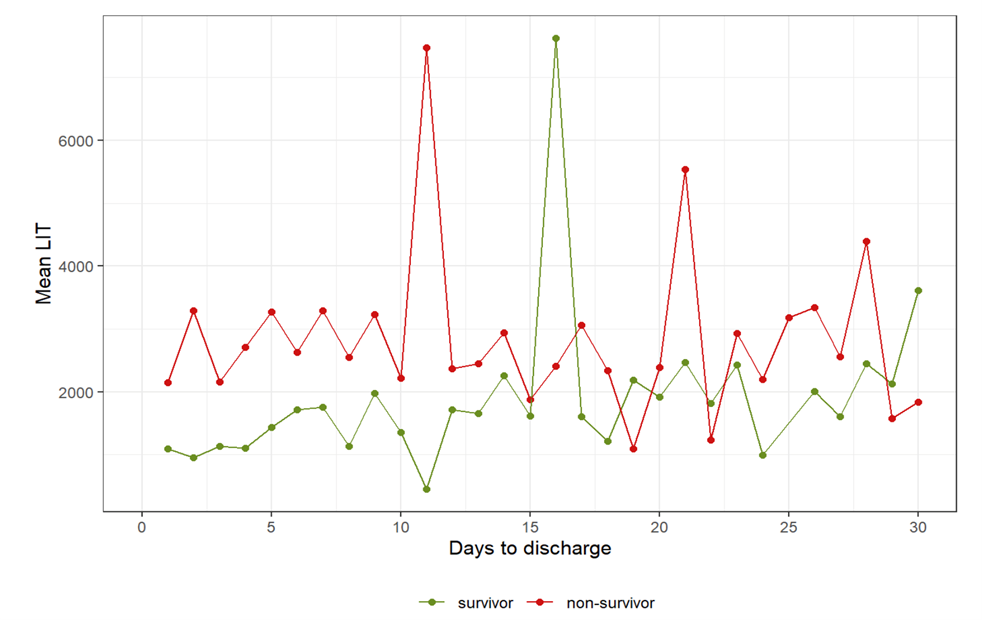


## Supplemental Figure 5. Trajectories of average LIT per number of days from discharge from hospital for survivor and non-survivor septic shock patients (up to 30 days length of stay)


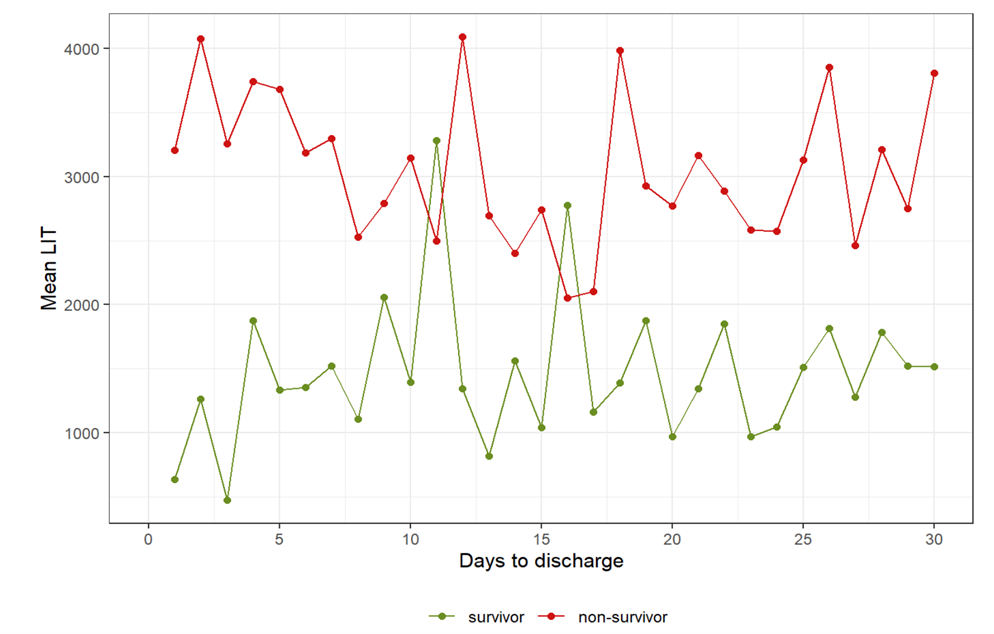

Supplement: Supplementary file 1 [file SupplementaryFile1.docx]
